# Impact of HIV on mortality among patients treated for tuberculosis in Lima, Peru: a prospective cohort study

**DOI:** 10.1186/s12879-016-1375-8

**Published:** 2016-02-01

**Authors:** Gustavo E. Velásquez, J. Peter Cegielski, Megan B. Murray, Martin J. A. Yagui, Luis L. Asencios, Jaime N. Bayona, César A. Bonilla, Hector O. Jave, Gloria Yale, Carmen Z. Suárez, Eduardo Sanchez, Christian Rojas, Sidney S. Atwood, Carmen C. Contreras, Janeth Santa Cruz, Sonya S. Shin

**Affiliations:** 1 Division of Infectious Diseases, Brigham and Women’s Hospital, Boston, MA USA; 2 Division of Infectious Diseases, Massachusetts General Hospital, Boston, MA USA; 3 Department of Medicine, Harvard Medical School, Boston, MA USA; 4 Division of Tuberculosis Elimination, Centers for Disease Control and Prevention, Atlanta, GA USA; 5 Department of Global Health and Social Medicine, Harvard Medical School, Boston, MA USA; 6 Department of Epidemiology, Harvard T.H. Chan School of Public Health, Boston, MA USA; 7 Division of Global Health Equity, Brigham and Women’s Hospital, Boston, MA USA; 8 Oficina General de Investigación y Transferencia Tecnológica, Instituto Nacional de Salud, Lima, Perú; 9 Departamento Académico de Medicina Preventiva y Salud Pública, Universidad Nacional Mayor de San Marcos, Lima, Perú; 10 Laboratorio Nacional de Referencia de Micobacterias, Instituto Nacional de Salud, Lima, Perú; 11 Health, Nutrition and Population, The World Bank Group, Washington DC, USA; 12 Estrategia Sanitaria Nacional de Prevención y Control de la Tuberculosis, Ministerio de Salud del Perú, Lima, Perú; 13 Dirección de Salud V Lima Ciudad, Programa de Control de Tuberculosis, Lima, Perú; 14 Dirección de Salud IV Lima Este, Programa de Control de Tuberculosis, Lima, Perú; 15 Servicio de Enfermedades Infecciosas y Tropicales, Hospital Nacional Hipólito Unanue, Lima, Perú; 16 Servicio de Neumología, Instituto Nacional Cardiovascular “Carlos Alberto Peschiera Carrillo”, Lima, Perú; 17 Partners In Health / Socios En Salud, Lima, Perú

**Keywords:** Tuberculosis, Human immunodeficiency virus, Clinical outcomes, Mortality, Prospective cohort study, Operational research

## Abstract

**Background:**

Human immunodeficiency virus (HIV)-associated tuberculosis deaths have decreased worldwide over the past decade. We sought to evaluate the effect of HIV status on tuberculosis mortality among patients undergoing treatment for tuberculosis in Lima, Peru, a low HIV prevalence setting.

**Methods:**

We conducted a prospective cohort study of patients treated for tuberculosis between 2005 and 2008 in two adjacent health regions in Lima, Peru (Lima Ciudad and Lima Este). We constructed a multivariate Cox proportional hazards model to evaluate the effect of HIV status on mortality during tuberculosis treatment.

**Results:**

Of 1701 participants treated for tuberculosis, 136 (8.0 %) died during tuberculosis treatment. HIV-positive patients constituted 11.0 % of the cohort and contributed to 34.6 % of all deaths. HIV-positive patients were significantly more likely to die (25.1 vs. 5.9 %, *P* < 0.001) and less likely to be cured (28.3 vs. 39.4 %, *P* = 0.003). On multivariate analysis, positive HIV status (hazard ratio [HR] = 6.06; 95 % confidence interval [CI], 3.96–9.27), unemployment (HR = 2.24; 95 % CI, 1.55–3.25), and sputum acid-fast bacilli smear positivity (HR = 1.91; 95 % CI, 1.10–3.31) were significantly associated with a higher hazard of death.

**Conclusions:**

We demonstrate that positive HIV status was a strong predictor of mortality among patients treated for tuberculosis in the early years after Peru started providing free antiretroviral therapy. As HIV diagnosis and antiretroviral therapy provision are more widely implemented for tuberculosis patients in Peru, future operational research should document the changing profile of HIV-associated tuberculosis mortality.

## Background

The global number of human immunodeficiency virus (HIV)-associated tuberculosis (TB) deaths has been decreasing since 2004 [1]. The epidemic of TB/HIV co-infection has prompted increased attention to and guidance for integrated delivery of HIV and TB services [2, 3]. Early antiretroviral therapy (ART) has been associated with improved survival among adults co-infected with HIV and TB in various settings [4–9].

In Peru, the prevalence of HIV among adults was estimated to be low at 0.5 % in 2005 [10]. However, only 1.9 % of all Peruvian TB patients had a known HIV status in 2005, and 1.2 % of known HIV-positive TB patients were on ART in 2010 [11]. During this period, Peru implemented aggressive tuberculosis control strategies, including rapid diagnosis and individualized regimens for MDR-TB [12, 13]. While there have been significant gains in diagnosis of HIV and ART provision for TB/HIV co-infected patients worldwide, particularly in high HIV prevalence settings, we sought to quantify the effect of HIV status on TB mortality in this low HIV prevalence setting. Our objective in the present study was to evaluate the impact of HIV on mortality in the early years after Peru started providing free ART, among a large prospective cohort of patients who underwent treatment for TB in two health regions of Lima, Peru.

## Methods

### Setting and enrollment

Between 2005 and 2010, the estimated incidence of TB in Peru was 140 and 106 cases per 100,000 population, respectively [11]. The HIV prevalence among Peruvian adults dropped during this period from 0.5 to 0.4 %, and patients co-infected with HIV comprised 1.9 to 2.6 % of notified TB cases [10, 11]. The enrollment methods for this cohort have been described previously [12, 14, 15]. In brief, we enrolled all patients with confirmed TB or presumptive TB with respiratory symptoms for at least 2 weeks who were living in 2 adjacent health regions in Lima (Lima Ciudad and Lima Este), and who met Peruvian National Tuberculosis Control Program (NTP) criteria for drug-susceptibility testing (DST) referral (Table 1) [13]. The criteria for DST referral detailed in Table 1 reflected a targeted testing strategy for patients at risk for multidrug-resistant TB (MDR-TB) [14]. Healthcare workers at local health establishments identified patients and sent their sputum samples to the reference laboratory for DST. Because all sputum samples for DST were sent to the district laboratories, we identified subjects eligible for enrollment by this referral. We enrolled patients at the time of sputum sample collection for culture and DST, from January 5, 2005 through March 5, 2008 in Lima Ciudad and from April 20, 2005 through May 27, 2008 in Lima Este. Study personnel visited each district laboratory on a regular basis to review sample referrals and confirm that all eligible subjects had been identified. There were no exclusion criteria for enrollment into the study.

**Table 1:** Peruvian National TB Control Program criteria for DST referral

A. Smear- or culture-positive patients at risk for MDR-TB without prior treatment history
Subjects may be referred for DST if they 1) are diagnosed with smear-positive pulmonary TB, 2) have no prior history of anti-tuberculosis treatment, and 3) have at least one of the following risk factors:
1. Household contact of patient with documented MDR-TB or presumptive MDR-TB (i.e., in treatment with second-line drugs, failed anti-tuberculosis treatment or died of TB in past 2 years)
2. HIV-positive by ELISA and Western Blot confirmation
3. Diabetes mellitus
4. Healthcare worker by profession, regardless of health care field, in the last 2 years
5. Student of health sciences in the past 2 years
6. Incarcerated or employee of the penitentiary system in the past 2 years
7. Chronic treatment with corticosteroids
8. Other condition of immunosuppression
9. Adverse reaction to TB medications that has required a change in regimen
10. Hospitalization for any indication in the past 2 years lasting more than 15 days
11. Presumptive treatment failure of Category I or II regimen (i.e., smear- or culture-positive between 2 and 4 months of treatment)
B. Patients who have received at least one previous course of treatment
Subjects may be referred for DST if they have any of the prior TB treatment histories:
1. Lost to follow-up for any previous regimen and now present for retreatment
2. Relapsed after completion of any previous regimen within less than 6 months
3. Treatment failed with any previous regimen
4. Received multiple courses of anti-tuberculosis treatment
5. Have a history of private or self-administered treatment
C. Confirmed or presumptive smear-negative TB among high-risk groups (tested by BACTEC™)
Subjects may be referred for DST if they 1) have presumptive or confirmed active pulmonary TB, 2) are smear-negative, and 3) have at least one of the following risk factors:
1. Pediatric household contact of patient with documented MDR-TB
2. Pediatric household contact of patient who has died of TB within the past 2 years
3. HIV-positive by ELISA and Western Blot confirmation

*DST* drug susceptibility testing, *ELISA* enzyme-linked immunosorbent assay, *HIV* human immunodeficiency virus, *MDR-TB* multidrug-resistant tuberculosis, *TB* tuberculosis

### Data collection

A trained team collected data prospectively from HIV and TB charts, laboratory registries, and databases using standardized forms. Baseline sociodemographic and clinical data, including baseline HIV status, were collected at enrollment. For HIV-positive patients, study personnel recorded the most recent CD4 cell count result and date, as well as receipt of ART, upon enrollment. HIV viral loads were not consistently available for this analysis. TB physicians reviewed all available chest x-rays (CXR) performed less than 1 year before and up to 1 month after enrollment, using standardized criteria for coding radiographic abnormalities. For each lung field, TB physicians recorded the presence of cavities, fibrosis, alveolar infiltrates, pneumothorax, pleural hemorrhage, nodules, disseminated/miliary disease, bullae, thoracic lymphadenopathy, and post-surgical changes. Data were entered into an Epi Info version 3.4.3 database (Centers for Disease Control and Prevention, Atlanta, GA, USA) and exported into an Access 2000 database (Microsoft Corporation, Redmond, WA, USA).

### Drug susceptibility testing methods

The DST methods for this cohort have been described previously [12, 14, 16, 17]. Under program conditions, the Peruvian National Reference Laboratory (NRL) performed mycobacterial culture and DST using the BACTEC™ 460 system (Becton, Dickinson and Company, Sparks, MD, USA) on paucibacillary and smear-negative samples produced by high-risk patients (HIV-positive, children, healthcare workers). Other smear-negative samples underwent mycobacterial culture on modified Ogawa medium followed by conventional DST for first-line drugs using the proportions method on Löwenstein-Jensen (LJ) medium at the district laboratory. Smear-positive samples were cultured on LJ medium and underwent DST using the indirect agar plate proportions method. Beginning in December 2005 in Lima Ciudad and March 2007 in Lima Este, these samples were cultured on modified LJ medium and tested for isoniazid and rifampicin resistance using the direct nitrate reductase assay (NRA) method [18]. All isolates found to be drug-resistant at the district laboratories were sent to the NRL for complete DST to first- and second-line drugs.

### HIV and tuberculosis management

In 2004, the Peruvian Ministry of Health began offering free ART through the Peruvian National HIV Program to patients with either 1) Stage III or Stage IV acquired immunodeficiency syndrome (AIDS) defining illnesses other than TB with CD4 count <350 cells/μL, or 2) Stage I or Stage II HIV disease with CD4 count <200 cells/μL according to World Health Organization criteria [19]. Peruvian national TB treatment guidelines recommended HIV screening for all TB patients [13]. HIV physicians evaluated HIV-positive patients monthly, with CD4 and viral load monitoring every 6 months. Peruvian national TB treatment guidelines made the following recommendations regarding timing of ART initiation for TB/HIV co-infected patients: 1) ART deferral until after completion of TB treatment for those with CD4 count >200 cells/μL; 2) ART initiation after the intensive phase of TB treatment for those with CD4 count 100–200 cells/μL; and 3) ART initiation as soon as feasible after TB treatment initiation for those with CD4 count <100 cells/μL (with no specific recommendation for early versus deferred ART) [13].

Details of the TB management have been published elsewhere [13, 20, 21]. New drug-susceptible TB (DS-TB) cases, regardless of HIV status, received a biphasic treatment regimen with first-line drugs isoniazid (H), rifampicin (R), ethambutol (E), and pyrazinamide (Z): 2RHZE/4(RH)_2_ [13]. Retreatment DS-TB cases received a regimen including streptomycin (S) in the intensive phase: 2RHZES/1RHZE/5(RHE)_2_ [13]. Patients with presumptive MDR-TB received empiric treatment with second-line therapy pending DST results: ethambutol, pyrazinamide, kanamycin, ciprofloxacin, ethionamide, cycloserine, and para-aminosalicylic acid [13]. The guidelines used to design individualized MDR-TB treatment regimens based on DST results have been described elsewhere [22]. All TB patients received directly observed treatment free of charge through the Peruvian NTP. We followed patients until a TB treatment outcome was recorded or until the administrative end of the study on June 26, 2010.

### Exposure variable definitions

We identified patients as having drug-resistant TB (DR-TB) if they had a positive culture for *M. tuberculosis* and DST results showed resistance to isoniazid and/or rifampicin. MDR-TB was defined as drug-resistance to at least isoniazid and rifampicin. Baseline microbiologic data was defined as smear, culture, and DST results from samples collected within 30 days of enrollment. If baseline drug resistance data were not available for isoniazid and rifampicin, the patient was considered to have no baseline DST result. Extrapulmonary TB was defined as TB localization in a non-pulmonary organ and/or evidence of pleural or disseminated (miliary) TB on CXR. Baseline HIV status was defined as positive if a positive HIV test result and/or receipt of ART was documented in the patient’s chart. Baseline HIV status was defined as negative if documented as such, or if the HIV status was unknown (either due to not being tested for HIV, or an unknown HIV test result). The standardized forms used to abstract data from clinical charts did not differentiate between unknown or negative HIV status, so we were unable to ascertain the proportion with unknown HIV status. Baseline CD4 cell count was defined as CD4 testing performed within 6 months of enrollment.

### Outcome variable definition

We followed patients from the time of enrollment to the date of TB treatment outcome, including death. The patients with no treatment outcome during the observation period were censored on the last day of follow-up. We excluded patients from the analysis if they were not treated for TB during the period of observation. TB treatment outcomes were defined according to previously published guidelines [23, 24].

### Statistical methods

We selected potential predictors of mortality from risk factors identified in the literature and clinical experience [9, 25–31], and evaluated them using univariate Cox proportional hazards models. We considered predictors associated with death on univariate analysis (*P* < 0.05) for inclusion in the multivariate Cox proportional hazards model evaluating the association between HIV and mortality. We built the multivariate model using a backward selection method, retaining age, gender, and predictors associated with death with a *P* < 0.20. Since HIV-positive patients tended to present with more severe clinical findings, we considered the possibility that the effect of HIV status on mortality during TB treatment was mediated by predictors significantly associated with death on univariate analysis (*P* < 0.05) that also corresponded to disease severity. We considered the following variables as potential mediators: ability to perform activities of daily living (ADLs), low body mass index (BMI), and dyspnea. We evaluated the direct effect of HIV status on time to death by comparing multivariate models with and without these variables. We tested the proportional hazards assumption in the multivariate models by using Schoenfeld residuals fitted to rank of analysis time, and we constructed a Kaplan-Meier survival curve stratified by HIV group. We also performed a secondary analysis in which we repeated the multivariate analyses while adjusting for baseline MDR-TB.

All analyses were performed using Stata/SE version 14.1 (StataCorp LP, College Station, TX, USA). The *χ*2 test or Fisher’s exact test was used to calculate *P* values, when appropriate. The Student’s *t* test was used for the two-sample mean-comparison test, when appropriate. All statistical tests were two-sided, and significance was set at α = 0.05.

### Ethics statement

Institutional review boards at the Partners Human Research Committee and the Peruvian National Institute of Health Committee of Research Ethics approved the original study protocol and the protocol amendment for this analysis. The original study was a programmatic effort under the leadership of the Peruvian National TB Control Program and the Peruvian National Institute of Health. An informed consent waiver was approved from both institutional review boards, because the data collection process required no contact with the patients; only included information that was routinely collected in the course of providing clinical care to patients; and the treatment regimens administered to patients were determined by the standards of care in Peru at the time, such that participation in the study did not adversely affect the rights and welfare of the subjects and patient confidentiality was maintained. The U.S. Centers for Disease Control and Prevention approved this activity as program evaluation and not human subjects research.

## Results

**Fig. 1:**
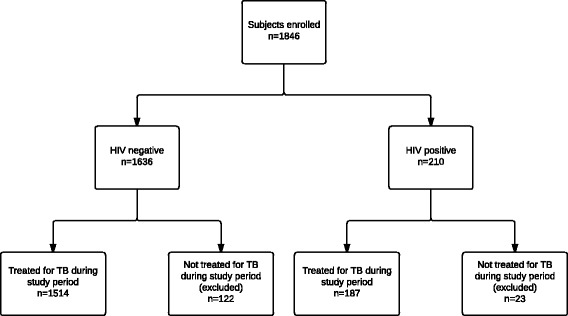
Study flow

Of the 1846 participants enrolled (Fig. 1), 145 (7.9 %) were excluded because they were not treated for TB during the observation period. The remaining 1701 participants were included in the analysis; 187 (11.0 %) were HIV-positive (Table 2). HIV-positive patients were more likely to be male, unemployed, and have a history of alcohol or illicit drug abuse compared with HIV-negative patients. HIV-positive patients tended to experience a shorter mean duration of TB symptoms before DST referral (2.5 ± 3.0 vs. 3.5 ± 4.9 months), but presented with more severe clinical findings such as weight loss, low BMI, dyspnea, and poor functional status in terms of ability to perform ADLs. They were also more likely to present with extrapulmonary TB and fewer pulmonary findings (abnormal CXR, cavitary disease, hemoptysis, sputum acid-fast bacilli (AFB) smear positivity).

**Table 2:** Characteristics of patients treated for tuberculosis, by HIV status*

Characteristic	HIV positive	HIV negative	Total
	(*N* = 187)	(*N* = 1514)	(*N* = 1701)
	No. (%)	No. (%)	No. (%)
Sociodemographic characteristics			
Age (years), mean ± SD	33.4 ± 10.6	33.6 ± 15.9	33.6 ± 15.4
Female gender	**49** (**26.2**)	**551** (**36.4**)	600 (35.3)
Married or lived together	64 (34.2)	554 (36.6)	618 (36.3)
Unemployed	**102** (**54.6**)	**535** (**35.3**)	637 (37.5)
Did not begin secondary level education, *n* = 1700	30 (16.0)	318 (21.0)	348 (20.5)
Tobacco use, *n* = 1699^a^	59 (31.9)	382 (25.2)	441 (26.0)
Alcohol abuse, *n* = 1699^b^	**85** (**46.0**)	**537** (**35.5**)	622 (36.6)
Illicit drug abuse, *n* = 1699^c^	**57** (**30.7**)	**252** (**16.7**)	309 (18.2)
Comorbidities			
Diabetes mellitus	**1** (**0.5**)	**182** (**12.0**)	183 (10.8)
Chronic corticosteroid therapy	0 (0)	6 (0.4)	6 (0.4)
Other immunosuppression	0 (0)	8 (0.5)	8 (0.5)
TB history and risk exposures			
Previously treated for TB	161 (86.1)	1326 (87.6)	1487 (87.4)
Prior incarceration, *n* = 1695^d^	17 (9.2)	106 (7.0)	123 (7.3)
Recent hospitalization for >15 days^d^	2 (1.1)	14 (0.9)	16 (0.9)
Healthcare occupational exposure^d,e^	**1** (**0.5**)	**84** (**5.6**)	85 (5.0)
Clinical presentation, all patients			
Duration of symptoms before DST solicited (months), mean ± SD, *n* = 1690	**2.5** ± **3.0**	**3.5** ± **4.9**	3.4 ± 4.8
Able to perform ADLs	**149** (**79.7**)	**1424** (**94.1**)	1573 (92.5)
Weight loss, *n* = 1698	**164** (**89.1**)	**1202** (**79.4**)	1366 (80.5)
BMI (kg/m^2^), mean ± SD, *n* = 1693	**19.9** ± **3.5**	**21.4** ± **3.6**	21.2 ± 3.6
Dyspnea, *n* = 1698	**49** (**26.3**)	**246** (**16.3**)	295 (17.4)
Hemoptysis, *n* = 1699	**3** (**1.6**)	**98** (**6.5**)	101 (5.9)
Chest radiography			
Abnormal CXR, *n* = 1651	**135** (**72.2**)	**1375** (**90.8**)	1510 (88.8)
Normal CXR, *n* = 1651	**41** (**21.9**)	**100** (**6.6**)	141 (8.3)
No CXR performed, *n* = 50	**11** (**5.9**)	**39** (**2.6**)	50 (2.9)
Cavitary disease, *n* = 1651	**27** (**15.3**)	**598** (**40.5**)	625 (37.9)
Site of TB disease			
Pulmonary only	**157** (**84.0**)	**1448** (**95.6**)	1605 (94.4)
Extrapulmonary with or without pulmonary	**30** (**16.0**)	**66** (**4.4**)	96 (5.6)
Clinical presentation, HIV positive patients			
Receiving ART at baseline	24 (12.8)	N/A	N/A
Receiving cotrimoxazole at baseline	29 (15.5)		
No CD4 performed	147 (78.6)	N/A	N/A
Baseline CD4 count (cells/μL) if performed, *n* = 40		N/A	N/A
< 100	13 (32.5)	N/A	N/A
100–350	23 (57.5)	N/A	N/A
> 350	4 (10.0)	N/A	N/A
Microbiologic data			
Baseline bacteriologically unconfirmed TB diagnosis^f^	**58** (**31.0**)	**51** (**3.4**)	109 (6.4)
Baseline sputum AFB smear positive, *n* = 1669	**86** (**50.3**)	**1362** (**90.9**)	1448 (86.8)
Baseline TB drug resistance status			
Drug susceptible	74 (39.6)	576 (38.0)	650 (38.2)
Mono-resistant (either H or R)	20 (10.7)	133 (8.8)	153 (9.0)
MDR-TB	**22** (**11.8**)	**386** (**25.5**)	408 (24.0)
No result	**71** (**38.0**)	**419** (**27.7**)	490 (28.8)
Treatment course			
Received second line drugs during study^g^	**36** (**19.3**)	**711** (**47.0**)	747 (43.9)
Non-adherence, *n* = 1689^h^	46 (24.6)	376 (25.0)	422 (25.0)

*ADLs* activities of daily living, *AFB* acid-fast bacilli, *ART* antiretroviral therapy, *BMI* body mass index, *CXR* chest x-ray, *DST* drug susceptibility test, *H* isoniazid, *HIV* human immunodeficiency virus, *MDR-TB* multidrug-resistant tuberculosis, *R* rifampicin, *SD* standard deviation, *TB* tuberculosis

*Values are presented as No. (%) unless otherwise specified. Boldface indicates statistically significant difference (*P* < 0.05) between the HIV positive and the HIV negative group

^a^Defined as current or past tobacco use of more than 10 packs/year

^b^Defined as current or past alcohol use that interfered with family, health, or work

^c^Defined as current or past illicit drug use that interfered with family, health, or work

^d^During the past 2 years

^e^Defined as healthcare worker or student

^f^Defined as any case with a positive baseline AFB (not paucibacillary) or with a positive baseline culture

^g^Defined as receiving a regimen containing second-line drugs at enrollment or post enrollment

^h^Defined as having spent >20 % of treatment regimen duration off drugs

Bacteriological confirmation and hence documented DST data were less frequently obtained among HIV-positive individuals. Among those with DST data, the proportion of MDR-TB was lower for HIV-positive versus HIV-negative individuals (11.8 vs. 25.5 %, *P* < 0.001). Among HIV-positive patients, 24 (12.8 %) were receiving ART at baseline, 29 (15.5 %) were receiving cotrimoxazole prophylaxis, and 147 (78.6 %) did not have baseline CD4 cell count data. Two (8.3 %) of the 24 HIV-positive patients who were receiving ART were also receiving cotrimoxazole at baseline. Of the 40 HIV-positive patients with baseline CD4 counts, 36 (90.0 %) had baseline CD4 lower than 350 cells/μL and 13 (32.5 %) were below 100 cells/μL.

**Fig. 2:**
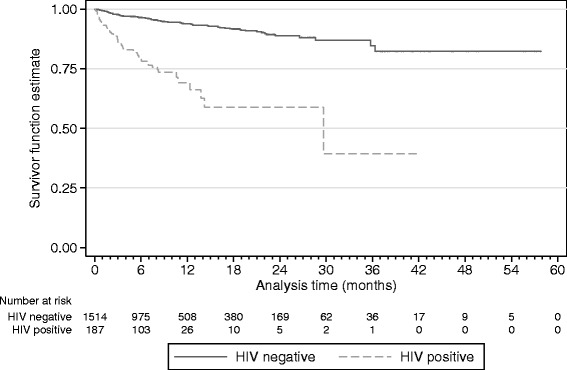
Survival curve for patients treated for tuberculosis, by HIV status

A total of 999 (58.7 %) patients were either cured or completed treatment (Table 3). HIV-positive patients were significantly more likely to die (25.1 vs. 5.9 %, *P* < 0.001) and less likely to be cured (28.3 vs. 39.4 %, *P* = 0.003). A Kaplan-Meier survival curve stratified by HIV group is shown in Fig. 2. The HIV-positive patients in the cohort contributed 47 (34.6 %) of all deaths; 42 (89.4 %) of deaths among the HIV-positive patients occurred among those who were not receiving ART at baseline. Among those who died, 6 (12.8 %) HIV-positive and 16 (18.0 %) HIV-negative patients had achieved culture conversion from positive to negative (*P* = 0.433, data not shown).

**Table 3:** Treatment outcomes of patients treated for tuberculosis, by HIV status*

Characteristic	HIV positive	HIV negative	Total	*P* value
	(*N* = 187)	(*N* = 1514)	(*N* = 1701)	
	No. (%)	No. (%)	No. (%)	
Favorable outcome	89 (47.6)	910 (60.1)	999 (58.7)	**0.001**
Cured	53 (28.3)	596 (39.4)	649 (38.2)	**0.003**
Treatment completed	36 (19.3)	314 (20.7)	350 (20.6)	0.635
Unfavorable outcome	85 (45.5)	464 (30.7)	549 (32.3)	<**0.001**
Default	37 (19.8)	345 (22.8)	382 (22.5)	0.353
Failure	1 (0.5)	30 (2.0)	31 (1.8)	0.245
Death	47 (25.1)	89 (5.9)	136 (8.0)	<**0.001**
Transferred out	10 (5.4)	105 (6.9)	115 (6.8)	0.415
Unknown	3 (1.6)	35 (2.3)	38 (2.2)	0.792

*HIV* human immunodeficiency virus

*Boldface indicates statistically significant difference (*P* < 0.05) between the HIV positive and the HIV negative group

The median duration of follow-up was 232 (interquartile range [IQR]: 159–486) days for a total of 568,602 person-days of follow-up, during which time there were 136 deaths. Among those who died, the median survival time was 111 (IQR: 53–263) days. In univariate analyses (Table 4), predictors associated with a lower hazard of death were female gender and ability to perform ADLs at baseline, while baseline characteristics associated with a higher hazard of death were unemployment, substance abuse, positive HIV status, recent hospitalization, weight loss, low BMI, dyspnea, and lack of bacteriological confirmation. We were unable to estimate the effect of CD4 categories on mortality given the high proportion of missing CD4 results among HIV-positive patients.

**Table 4:** Univariate predictors associated with time to death among patients treated for tuberculosis*

Characteristic	Unadjusted HR (95 % CI)	*P* value
Sociodemographic characteristics		
Pediatric case^a^	0.36 (0.12–1.14)	0.082
Female gender	**0.65** (**0.45**–**0.95**)	**0.025**
Unemployed	**2.95** (**2.09**–**4.18**)	<**0.001**
Did not begin secondary level education, *n* = 1700	1.08 (0.71–1.65)	0.712
Substance abuse, *n* = 1699^b^	**1.72** (**1.23**–**2.41**)	**0.002**
Comorbidities		
HIV positive	**5.99** (**4.18**–**8.59**)	<**0.001**
Diabetes mellitus	0.57 (0.26–1.22)	0.146
TB history and risk exposures		
Previously treated for TB	1.62 (0.90–2.93)	0.110
Prior incarceration, *n* = 1695^c^	1.07 (0.52–2.19)	0.854
Recent hospitalization for >15 days^c^	**4.13** (**1.53**–**11.2**)	**0.005**
Healthcare occupational exposure^c,d^	0.15 (0.02–1.08)	0.060
Clinical presentation, all patients		
Duration of symptoms >3 months before DST solicited, *n* = 1690	0.99 (0.70–1.41)	0.970
Weight loss, *n* = 1698	**2.99** (**1.65**–**5.43**)	<**0.001**
Able to perform ADLs	**0.15** (**0.10**–**0.21**)	<**0.001**
Low BMI, *n* = 1693^e^	**2.38** (**1.70**–**3.35**)	<**0.001**
Dyspnea, *n* = 1698	**2.90** (**2.04**–**4.11**)	<**0.001**
Hemoptysis, *n* = 1699	0.52 (0.21–1.26)	0.147
No CXR performed	1.59 (0.65–3.88)	0.312
Abnormal CXR, *n* = 1651	0.60 (0.35–1.02)	0.061
Cavitary disease, *n* = 1651	0.87 (0.61–1.24)	0.446
Extrapulmonary TB	1.51 (0.82–2.80)	0.188
Clinical presentation, HIV positive patients only		
Receiving ART at baseline, *n* = 187	0.72 (0.28–1.82)	0.482
Receiving cotrimoxazole at baseline, *n* = 187	0.53 (0.21–1.36)	0.188
No CD4 performed, *n* = 187	1.25 (0.60–2.61)	0.550
Microbiologic data		
Baseline bacteriologically unconfirmed TB diagnosis^f^	**2.02** (**1.14**–**3.59**)	**0.016**
Baseline sputum AFB smear positive, *n* = 1669	0.87 (0.53–1.43)	0.589
Baseline MDR-TB, *n* = 1211	1.10 (0.73–1.65)	0.658
No baseline DST result	1.02 (0.70–1.51)	0.906
Treatment course		
Received second line drugs during study^g^	0.84 (0.57–1.23)	0.376
Non-adherence, *n* = 1689^h^	0.84 (0.56–1.25)	0.381

*ADLs* activities of daily living, *AFB* acid-fast bacilli, *ART* antiretroviral therapy, *BMI* body mass index, *CI* confidence interval, *CXR* chest x-ray, *DST* drug susceptibility test, *HIV* human immunodeficiency virus, *HR* hazard ratio; *MDR-TB* multidrug-resistant tuberculosis, TB tuberculosis

*Boldface indicates *P* < 0.05. For covariates with full data, the model included *n* = 1701 (568,602 person-days, 136 deaths). For covariates with missing data, the *n* for the model is provided

^a^Defined as age <15 years

^b^Defined as current or past alcohol use or illicit drug use that interfered with family, health, or work

^c^During the past 2 years

^d^Defined as healthcare worker or student

^e^Defined as BMI <20 kg/m^2^ in men, BMI <18.5 kg/m^2^ in women

^f^Defined as any case with a positive baseline AFB (not paucibacillary) or with a positive baseline culture

^g^Defined as receiving a regimen containing second-line drugs at enrollment or post enrollment

^h^Defined as having spent >20 % of treatment regimen duration off drugs

On multivariate analysis, we used 1699 (99.9 %) observations with complete data to build the multivariate model (Table 5). Positive HIV status, unemployment, and baseline sputum AFB positivity were all significantly associated with a higher hazard of death. When we adjusted for baseline ability to perform ADLs, low BMI, and dyspnea, we observed that the direct effect of positive HIV status on hazard of death was 22.9 % lower than the marginal effect (hazard ratio [HR] = 4.67 vs. HR = 6.06, respectively). For the secondary analysis, we repeated the multivariate analyses adjusting for baseline MDR-TB, using 1211 (71.2 %) observations with complete DST data to build the multivariate model (Table 6). Baseline MDR-TB showed a trend towards a higher hazard of death in this cohort, however the association was not statistically significant (HR = 1.40; 95 % confidence interval [CI], 0.92–2.13). We observed that positive HIV status and unemployment remained significantly associated with a higher hazard of death, while the effect of baseline sputum AFB positivity was no longer significant (HR = 2.05; 95 % CI, 0.96–4.36; *P* = 0.063). Adjusting for potential mediators, the direct effect of positive HIV status on hazard of death was 25.9 % lower than the marginal effect (HR = 4.71 vs. HR = 6.36, respectively).

**Table 5:** Multivariate model examining association between HIV infection and time to death among patients treated for tuberculosis*

Characteristic	Multivariate model		Direct effect model	
	(without mediators)		(with mediators)	
	*N* = 1699, 136 deaths		*N* = 1658, 127 deaths	
	Adjusted HR		Adjusted HR	
	(95 % CI)	*P* value	(95 % CI)	*P* value
HIV positive	**6.06** (**3.96**–**9.27**)	<**0.001**	**4.67** (**2.99**–**7.29**)	<**0.001**
Pediatric case^a^	0.64 (0.15–2.64)	0.536	0.45 (0.11–1.90)	0.276
Female gender	0.92 (0.61–1.37)	0.670	0.96 (0.63–1.47)	0.870
Unemployed	**2.24** (**1.55**–**3.25**)	<**0.001**	**1.82** (**1.24**–**2.65**)	**0.002**
Baseline sputum AFB smear positive	**1.91** (**1.10**–**3.31**)	**0.021**	**1.88** (**1.07**–**3.29**)	**0.028**
Able to perform ADLs			**0.26** (**0.17**–**0.41**)	<**0.001**
Low BMI^b^			**1.71** (**1.18**–**2.48**)	**0.004**
Dyspnea			**1.56** (**1.04**–**2.34**)	**0.030**

*ADLs* activities of daily living, *AFB* acid-fast bacilli, *BMI* body mass index, *CI* confidence interval, *HIV* human immunodeficiency virus, *HR* hazard ratio

*Boldface indicates *P* < 0.05. The total *n* for each multivariate model is lower than the analysis cohort (*n* = 1701) because of complete case drop of observations with missing data

^a^Defined as age <15 years

^b^Defined as BMI <20 kg/m^2^ in men, BMI <18.5 kg/m^2^ in women

**Table 6:** Multivariate model examining association between HIV infection and time to death among patients treated for tuberculosis, adjusted for baseline MDR-TB*

Characteristic	Multivariate model		Direct effect model	
	(without mediators)		(with mediators)	
	*N* = 1211, 101 deaths		*N* = 1192, 97 deaths	
	Adjusted HR		Adjusted HR	
	(95 % CI)	*P* value	(95 % CI)	*P* value
HIV positive	**6.36** (**3.98**–**10.2**)	<**0.001**	**4.71** (**2.87**–**7.73**)	<**0.001**
Pediatric case^a^	0.41 (0.06–3.01)	0.381	0.32 (0.04–2.37)	0.265
Female gender	0.77 (0.48–1.25)	0.288	0.84 (0.51–1.38)	0.484
Unemployed	**1.95** (**1.28**–**2.97**)	**0.002**	**1.61** (**1.05**–**2.47**)	**0.029**
Baseline sputum AFB smear positive	2.05 (0.96–4.36)	0.063	2.14 (1.00–4.61)	0.051
Baseline MDR-TB	1.40 (0.92–2.13)	0.121	1.37 (0.89–2.10)	0.150
Able to perform ADLs			**0.27** (**0.16**–**0.45**)	<**0.001**
Low BMI^b^			**2.09** (**1.37**–**3.19**)	**0.001**
Dyspnea			**1.27** (**0.79**–**2.07**)	**0.326**

*ADLs* activities of daily living, *AFB* acid-fast bacilli, *BMI* body mass index, *CI* confidence interval, *HIV* human immunodeficiency virus, *HR* hazard ratio, *MDR-TB* multidrug-resistant tuberculosis

*Boldface indicates *P* < 0.05. The total *n* for each multivariate model is lower than the analysis cohort (*n* = 1701) because of complete case drop of observations with missing data

^a^Defined as age <15 years

^b^Defined as BMI <20 kg/m^2^ in men, BMI <18.5 kg/m^2^ in women

## Discussion

We describe the impact of positive HIV status on mortality in a cohort of 1701 patients treated for TB in two health regions in Lima, Peru, demonstrating that HIV-positive patients accounted for 34.6 % of deaths and had a six-fold higher hazard of death during TB treatment when compared to HIV-negative patients. HIV-positive patients presented with more severe clinical findings than HIV-negative patients; we found that some of the effect of HIV status on mortality was mediated by ability to perform ADLs, low BMI, and dyspnea at the time of enrollment. After adjusting for these markers of baseline disease severity, the direct effect of HIV status on mortality remained significantly associated with a more than four-fold higher hazard of death. In the secondary analysis, the observed effect of positive HIV status on mortality was similar when we adjusted for baseline MDR-TB. Our results are consistent with prior studies showing that HIV is a predictor of poor TB treatment outcomes in Peru [26, 27, 32–35], and serves to highlight the need for improved management of TB/HIV co-infection in this low HIV prevalence setting. To our knowledge, this is the largest published prospective cohort evaluating mortality among TB/HIV co-infected patients in Peru.

Our finding that 78.6 % of HIV-positive patients had missing CD4 cell count data suggested that the majority of HIV patients were not receiving adequate HIV care at the time of enrollment. This HIV treatment gap is also reflected in publicly reported data from Peru, where 1.9 % of all Peruvian TB patients had a known HIV status in 2005 and 1.2 % of known HIV-positive TB patients were on ART in 2010 [11]. We observed that 42 (89.4 %) of deaths among the HIV-positive patients in this cohort occurred among those who were not receiving ART at baseline. Since early ART has been associated with improved survival among adults co-infected with HIV and TB in other settings [4–9], expanded access to early ART and retention in care could have prevented a sizable proportion of the mortality in this cohort.

This large cohort of patients was enrolled and followed prospectively under real program conditions. Since HIV-positive status was an indication for DST among TB patients in Peru, our sampling strategy overrepresented HIV-positive patients who constituted 11.0 % of the analysis cohort. We classified the HIV-negative group as those patients with either documented negative or unknown HIV status. Although the standardized forms we used to abstract data from clinical charts did not differentiate between unknown or negative HIV status, we consider this a reasonable assumption in Peru, where only 1.9–2.6 % of notified TB cases were documented to be HIV-positive during the study period [11]. We would expect that nondifferential misclassification of HIV status would bias the effect of HIV status on mortality towards the null. However, this misclassification may diminish the ability of future studies to document a reduction in the magnitude of the effect of HIV status on mortality, when compared to our estimates.

We found that baseline MDR-TB was associated with a non-significant trend towards higher hazard of death in this cohort. This may have been due to an existing aggressive TB control strategy in Peru, including rapid diagnosis and individualized regimens for MDR-TB. We have previously published data from this cohort suggesting that the concurrent implementation of rapid DST in Peru was associated with favorable treatment outcome and prolonged survival among individuals without previous anti-TB treatment, and a non-significant trend towards improved outcomes among those with drug-resistant TB [15]. Despite the observational nature of this study, frequent visits by our study personnel to health centers could have sensitized healthcare workers to follow screening and treatment protocols more closely than they would have otherwise.

We also found that 12.8 % of deaths in the HIV-positive group and 18.0 % of deaths in the HIV-negative group had achieved culture conversion from positive to negative. A limitation in this study is that our prospective clinical chart reviews did not capture causes of death. We were therefore unable to ascertain whether deaths in this cohort were truly due to TB or due to competing risks in this urban population in Lima, for example from comorbidities, accidental death, or violence. Deaths among those who had achieved culture conversion may have biased the effect of HIV status on mortality away from the null if in fact these patients died from non-TB causes. Deaths among those who were not treated for TB, an exclusion criterion for this analysis, may have biased the effect of HIV status on mortality in either direction.

## Conclusions

We observed a strong influence of HIV status on mortality within the context of a National TB Program delivering rapid diagnosis and individualized regimens for MDR-TB, but with challenges providing HIV care to co-infected patients. These findings suggest that aggressive strategies for TB alone were not sufficient: universal HIV testing and timely care for those living with HIV were also needed [36]. Future operational research will be important to establish the changing profile of HIV-associated TB mortality in Peru, as HIV diagnosis and ART provision are more widely implemented.

## Abbreviations

ADLs: activities of daily living
AFB: acid-fast bacilli
AIDS: acquired immunodeficiency syndrome
ART: antiretroviral therapy
BMI: body mass index
CI: confidence interval
CXR: chest x-ray
DR-TB: drug-resistant tuberculosis
DST: drug-susceptibility testing
DS-TB: drug-susceptible tuberculosis
E: ethambutol
H: isoniazid
HIV: human immunodeficiency virus
HR: hazard ratio
IQR: interquartile range
LJ: Löwenstein-Jensen
MDR-TB: multidrug-resistant tuberculosis
NRA: nitrate reductase assay
NRL: National Reference Laboratory
NTP: National Tuberculosis Control Program
R: rifampicin
S: streptomycin
TB: tuberculosis
Z: pyrazinamide

## References

[1] World Health Organization (2014). Global tuberculosis report 2014.

[2] World Health Organization (2004). Interim policy on collaborative TB/HIV activities.

[3] World Health Organization (2012). WHO policy on collaborative TB/HIV activities: guidelines for national programmes and other stakeholders.

[4] Abdool Karim SS, Naidoo K, Grobler A, Padayatchi N, Baxter C, Gray AL (2011). Integration of antiretroviral therapy with tuberculosis treatment. N Engl J Med.

[5] Blanc F-X, Sok T, Laureillard D, Borand L, Rekacewicz C, Nerrienet E (2011). Earlier versus later start of antiretroviral therapy in HIV-infected adults with tuberculosis. N Engl J Med.

[6] Havlir DV, Kendall MA, Ive P, Kumwenda J, Swindells S, Qasba SS (2011). Timing of antiretroviral therapy for HIV-1 infection and tuberculosis. N Engl J Med.

[7] Török ME, Yen NTB, Chau TTH, Mai NTH, Phu NH, Mai PP (2011). Timing of initiation of antiretroviral therapy in human immunodeficiency virus (HIV)--associated tuberculous meningitis. Clin Infect Dis.

[8] Manosuthi W, Mankatitham W, Lueangniyomkul A, Thongyen S, Likanonsakul S, Suwanvattana P (2012). Time to initiate antiretroviral therapy between 4 weeks and 12 weeks of tuberculosis treatment in HIV-infected patients: results from the TIME study. J Acquir Immune Defic Syndr.

[9] Marcy O, Laureillard D, Madec Y, Chan S, Mayaud C, Borand L (2014). Causes and determinants of mortality in HIV-infected adults with tuberculosis: an analysis from the CAMELIA ANRS 1295-CIPRA KH001 randomized trial. Clin Infect Dis.

[10] UNAIDS: AIDSinfo. Peru. [http://aidsinfo.unaids.org/]

[11] World Health Organization (2013). Global tuberculosis report 2013.

[12] Shin SS, Yagui MJA, Asencios L, Yale G, Suárez CZ, Quispe N (2008). Scale-up of multidrug-resistant tuberculosis laboratory services, Peru. Emerg Infect Dis.

[13] Norma Técnica De Salud Para El Control De La Tuberculosis. Lima: Estrategia Sanitaria Nacional para la Prevención y Control de la Tuberculosis, Dirección General de Salud de las Personas, Ministerio de Salud; 2006.

[14] Velásquez GE, Yagui MJA, Cegielski JP, Asencios L, Bayona J, Bonilla C (2011). Targeted drug-resistance testing strategy for multidrug-resistant tuberculosis detection, Lima, Peru, 2005–2008. Emerg Infect Dis.

[15] Shin SS, Asencios L, Yagui MJA, Yale G, Suárez CZ, Bayona J (2012). Impact of rapid drug susceptibility testing for tuberculosis: program experience in Lima, Peru. Int J Tuberc Lung Dis.

[16] Asencios L, Yale G, Yagui MJA, Quispe N, Taylor A, Blaya JA (2008). Programmatic implementation of rapid DST for Mycobacterium tuberculosis in Peru. Int J Tuberc Lung Dis.

[17] Roberts GD, Goodman NL, Heifets L, Larsh HW, Lindner TH, McClatchy JK (1983). Evaluation of the BACTEC radiometric method for recovery of mycobacteria and drug susceptibility testing of Mycobacterium tuberculosis from acid-fast smear-positive specimens. J Clin Microbiol.

[18] Asencios L, Sloutsky A, Stowel M (2012). Método De Nitrato-Reductasa (GRIESS) Para La Detección Rápida De La Susceptibilidad a Isoniacida Y Rifampicina.

[19] World Health Organization (2004). Scaling Up antiretroviral therapy in resource-limited settings: treatment guidelines for a public health approach, 2003 revision.

[20] Blaya JA, Shin SS, Yagui MJA, Yale G, Suárez CZ, Asencios L (2007). A web-based laboratory information system to improve quality of care of tuberculosis patients in Peru: functional requirements, implementation and usage statistics. BMC Med Inform Decis Mak.

[21] Mitnick CD, Bayona JJ, Palacios EE, Shin SS, Furin JJ, Alcántara FF (2003). Community-based therapy for multidrug-resistant tuberculosis in Lima, Peru. N Engl J Med.

[22] Mukherjee JS, Rich ML, Socci AR, Joseph JK, Virú FA, Shin SS (2004). Programmes and principles in treatment of multidrug-resistant tuberculosis. Lancet.

[23] World Health Organization (2010). Treatment of tuberculosis: guidelines.

[24] Laserson KF, Thorpe LE, Leimane V, Weyer K, Mitnick CD, Riekstina V (2005). Speaking the same language: treatment outcome definitions for multidrug-resistant tuberculosis. Int J Tuberc Lung Dis.

[25] Shin SS, Pasechnikov AD, Gelmanova IY, Peremitin GG, Strelis AK, Mishustin S (2006). Treatment outcomes in an integrated civilian and prison MDR-TB treatment program in Russia. Int J Tuberc Lung Dis.

[26] Kawai V, Soto G, Gilman RH, Bautista CT, Caviedes L, Huaroto L (2006). Tuberculosis mortality, drug resistance, and infectiousness in patients with and without HIV infection in Peru. Am J Trop Med Hyg.

[27] Bernabé-Ortiz A (2008). Factors associated with survival of patients with tuberculosis in Lima, Peru. Rev Chilena Infectol.

[28] Duarte EC, Bierrenbach AL, da Silva JB, Tauil PL, de Fátima Duarte E (2009). Factors associated with deaths among pulmonary tuberculosis patients: a case–control study with secondary data. J Epidemiol Community Health.

[29] Getahun B, Ameni G, Biadgilign S, Medhin G (2011). Mortality and associated risk factors in a cohort of tuberculosis patients treated under DOTS programme in Addis Ababa, Ethiopia. BMC Infect Dis.

[30] Kliiman K, Altraja A (2010). Predictors and mortality associated with treatment default in pulmonary tuberculosis. Int J Tuberc Lung Dis.

[31] Lefebvre N, Falzon D (2008). Risk factors for death among tuberculosis cases: analysis of European surveillance data. Eur Respir J.

[32] Kurbatova EV, Taylor A, Gammino VM, Bayona J, Becerra MC, Danilovitz M (2012). Predictors of poor outcomes among patients treated for multidrug-resistant tuberculosis at DOTS-plus projects. Tuberculosis (Edinb).

[33] Palacios E, Franke M, Muñoz M, Hurtado R, Dallman R, Chalco K (2012). HIV-positive patients treated for multidrug-resistant tuberculosis: clinical outcomes in the HAART era. Int J Tuberc Lung Dis.

[34] Périssé ARS, Smeaton L, Chen Y, La Rosa A, Walawander A, Nair A (2013). Outcomes among HIV-1 infected individuals first starting antiretroviral therapy with concurrent active TB or other AIDS-defining disease. PLoS ONE.

[35] Chung-Delgado K, Guillen-Bravo S, Revilla-Montag A, Bernabé-Ortiz A (2015). Mortality among MDR-TB cases: comparison with drug-susceptible tuberculosis and associated factors. PLoS ONE.

[36] Sebastian JL, Bonilla C, Lecca LW, Contreras C, Muñoz M, Palacios E (2008). Community-based liaisons to improve referral and access to HIV treatment for TB co-infected patients in Peru.

